# The Prevention of Mental Disorders has a Bright Future

**DOI:** 10.3389/fpubh.2014.00060

**Published:** 2014-06-04

**Authors:** Vladeta Ajdacic-Gross

**Affiliations:** ^1^Department of Psychiatry, Psychotherapy and Psychosomatics, University Hospital of Psychiatry Zurich, University of Zurich, Zurich, Switzerland

**Keywords:** prevention, mental disorders, public mental health, risk factors

## Abstract

This article takes four looks at the status of prevention in psychiatry. The first glance is critical, shaped by disappointment at the slow progress in the understanding of psychiatric diseases and the lack of promise in prevention. The second look is less humble. It characterizes and acknowledges the efforts made so far. The third and the fourth perspectives optimistically announce a new age in research and prevention. Breakthroughs, whose contours are already appearing on the horizon today, will transform the prevention of psychiatric diseases into a success story within the next 10–15 years.

## A Critical First Glance

There are a great many arguments that can lead us to doubt the prospects of psychiatric research and therapy:

- the conflicting discussions on the number of diagnoses implemented in the new version of the Diagnostic and Statistical Manual of Mental Disorders (1): overtly anachronistic if psychiatry were a developing discipline;
- the continuing failure to understand the etiopathogenesis of psychiatric diseases – the terms psychiatric diseases and mental disorders are used interchangeably – despite progressing basic research in neurosciences and the availability of myriads of empirical results;
- the difficulties in supplementing categorical with dimensional concepts in disease models and thinking about psychiatric diagnoses (2);
- the lack of major discoveries and innovations in research, therapies, and drug development in recent decades (3);
- the missionary impetus of new and highly redundant concepts related to positive mental health such as locus of control, self-efficacy, hardiness, sense of coherence, emotional intelligence, resilience, coping etc.

In prevention, the disastrous state of affairs is primarily mirrored by the lack of primary – in particular, universal – prevention efforts. Disappointment is apparent in the 2001 WHO report (4), which stated (p. 64): “Currently, there is no evidence that interventions proposed for primary prevention of depression are effective except in a few isolated studies. Currently, primary prevention of schizophrenia is not possible. The precise etiology of the hyperkinetic disorders – hyperactivity in children, often with involuntary muscular spasms – is unknown, thus primary prevention is currently not possible.”

In the meantime, further WHO reports (5–7) and reports from other organizations have been published. Many setting- and group-specific prevention projects have been initiated. However, there is still no sufficient knowledge about specific risk factors and specific etiopathogenic mechanisms that could be implemented in specific prevention measures. Most of the known biopsychosocial risk factors are generic and apply to many neuropsychiatric and psychiatric diseases (Table 1).

**Table 1:** **Risk factors for mental disorders**.

Pregnancy/delivery
Infectious diseases
Substance abuse
Famine/poor diet
Complications during pregnancy
Complications during delivery
Preterm delivery, low/high birth weight
Violence, deprivation during childhood
Abuse, violence (emotional, physical)
Neglect (emotional, physical)
Quarrels between parents, close others
Other adversities in childhood
Broken home
Substance abuse of parents
Quarrels with other children, bullying
Problems with teacher, fear of school
Other violence
Violence, bullying in family
Violence to close others
Bullying in childhood, adolescence, adulthood, at work
Sexual abuse
Being the victim of violence in a public space
Substance abuse in adolescence, adulthood
Cannabis
Smoking
Alcohol
Illicit drugs
Poly-drug use
Traumatic events
Serious somatic illness, stroke
War, natural disaster, etc.
Accidents
Loss of loved ones (e.g., through suicide, bereavement, separation/divorce)
Chronic distress
Chronic pain
No control over stressors
Burnout/boreout
Care of demented family members, geriatric, or long-term care
Hormonally driven symptoms
Menstruation-related symptoms
Postpartum symptoms
Infectious diseases
Symptoms related to postinfectious fatigue or depression
Infectious diseases during pregnancy/childhood/adulthood
Not unequivocally referable or reciprocal risk factors
Poor diet
Sleep problems
Urbanicity
Migration

While some of the risk factors (physical and sexual abuse, bullying, maternal infections during pregnancy) would be effectively approachable via primary prevention measures, this is difficult for other risk factors (unemployment, loss of loved ones, etc.), where prevention can hardly overcome the limitation of being a drop in the bucket. Behavioral changes in the population, either culturally or socioeconomically shaped, easily overshadow any well-intended efforts.

Nevertheless, given the profile of most psychiatric diseases – young age at onset, chronic course, high levels of comorbidity – effective primary prevention measures would be extraordinarily beneficial. Meanwhile, there is no intentional, thoughtful universal prevention addressing large groups of the population or the population as a whole.

To make matters worse, public mental health (and thereby implicitly the prevention of psychiatric diseases) is subject to norms, hopes, and targets developed in public health, which in their turn have emerged from highly developed and successful subdisciplines dealing with infectious diseases, cardiovascular diseases (CVDs), or cancer. What would public health look like if it cared about the most difficult issues, for example autoimmune or neurological diseases, i.e., complex diseases with a largely unknown etiology, pure therapeutic opportunities, early age at onset, and a typically chronic course? This rhetorical question makes clear that the perception of what public health is and what prevention should do, is highly skewed. Focusing on diseases of old age and a couple of acute diseases is not the main scope of public mental health or of public health related to autoimmunity and neurology.

## A Second Glance: Promising Efforts

**Figure 1:**
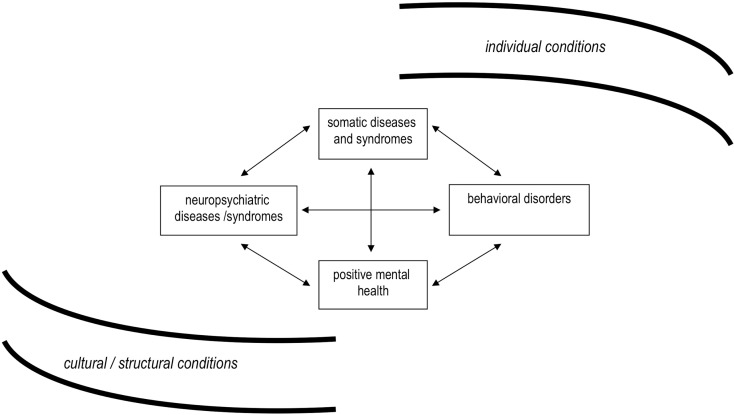
**Components of a general model of health / public health**.

However, things are too complex to be desperate. Basically, we need to distinguish four fields of interest (Figure 1). Apart from somatic and mental health, behavioral disorders and positive health are also included. Notably, the relations of these components are at least as specific as the components themselves.

The model shows at a glance that prevention of mental disorders involves not only diagnoses and symptoms but at least also behaviors such as suicide, substance use, and others that are related to somatic functions (sleep, eating, etc.). There are basic differences between the prevention of mental disorders and the prevention of behavioral disorders. Typically, the latter offer more opportunities than the former, and promise to be more successful. Suicide is an interesting example for several reasons.

A recent noteworthy aspect of suicide is the unexpected 20–40% decrease in rates in many Western countries over recent decades. This not only offers challenging opportunities to learn about underlying mechanisms but also indicates that behavioral disorders are rarely static, often undergo change, and thus make prevention efforts promising.

Furthermore, suicide can serve as a model for the utility of differentiating prevention domains in behavioral disorders:

- methods/means of suicide
- imitation suicides (media, other sources of information, school classes)
- risk groups, persons at increased risk
- professionals
- information/education (mental health literacy, knowledge about institutions and professionals providing help, problem-solving competence, etc.)
- easily accessible support facilities, helplines (for both persons with increased risk and professionals)
- available inpatient and outpatient mental health services
- developed emergency medicine.

In terms of these domains, the recent decrease in suicide rates has been attributed to increasing prescription rates of antidepressants, that is, to improved services (8). While some authors attribute this development to the availability of selective serotonin reuptake inhibitors (SSRIs), it seems no less realistic in the era of stress and burnout that the help-seeking behavior of young and better educated persons has distinctly changed – and thus also the demand for services.

The spectrum of relevant prevention domains may diverge among distinct behavioral disorders. However, approaching means and behaviors always remains an additional option apart from addressing emotions and mood. Most importantly, prevention measures here also include primary and universal prevention. The measures can be both indirect and direct, i.e., address the target behavior specifically. The case of suicide shows that prevention has more levers in some areas of public mental health than in others.

The second glance at current prevention measures also reveals a complex picture regarding mental disorders and additionally dampens the initial disappointment. First of all, there are many measures that cover primary prevention but are indirect. This means that they target a particular issue and *en passant* involve – intended or unintended – beneficial effects for mental health as well:

- violence prevention
- injury prevention
- prevention of other traumatic events
- prenatal health, coaching, and care of pregnant mothers
- prevention of substance use and infectious diseases (rubella, *Toxoplasma gondii*) during pregnancy
- counseling in families at risk
- coaching of adolescent mothers
- prevention of infectious diseases known to increase the risk for psychiatric diseases
- substance abuse and addictive behavior prevention
- voluntary community work, social support, neighborhood networks
- projects and measures addressing health promotion, health literacy, healthy behaviors

Currently available direct prevention measures or projects are often related to specific settings, for example, school, university, military, work settings, health services (9). Again, there are universal, selective, and indicated approaches, particularly heterogeneous in the school setting (10) and, besides, shown to reduce for example the depression incidence by 25% (11). The effect sizes can be expected to be different between the prevention approaches, i.e., higher in selective and indicated prevention, however, the outcomes are not unambiguous (12). A major problem in school-based universal prevention programs is that the effects appear to evaporate in the medium and long run.

How broad the spectrum of prevention approaches (universal, selective, indicated) is not only dependent on the settings but also on the disorders targeted. The earlier a disorder starts and the more frequent it is, the more pressure increases to begin prevention early in life and to use all accessible tools. This applies not only to anxiety disorders (13), but also to mood disorders (14). In contrast, efforts in the prevention of borderline personality disorder (15), psychosis, and schizophrenia (16, 17) are mostly limited to selective prevention (addressing persons at risk) or to indicated prevention (addressing persons with symptoms or subthreshold diagnoses) even though the potential for universal prevention should not be underestimated (18, 19).

A second large group of prevention measures is integrated in regional or national campaigns located at the borderline between primary and secondary prevention (20). One major direction is to fight stigma of mental disorders [see, for example the “time to change” campaign (21)], not only to improve the social status of mentally ill persons but also to encourage people to seek help early when encountering mental problems. Another major direction is to improve knowledge (mental health literacy) and to change attitudes toward mental health and mental illness in general. An example of such a campaign is the Nuremberg alliance against depression (22), which follows several aims at once within a concerted action plan: training and support of family doctors, public relations campaigns providing information about depression, cooperation with professionals and local media, and support for self-help activities and for high-risk groups.

The Nuremberg campaign is particularly intriguing because it shows that population-related prevention programs can be successful and that their success depends mainly on two components:

- the coordinated implementation of several synergistic actions addressing different groups (in this instance lay persons, affected persons, physicians, professionals);
- the need for continuing actions at lower levels after a major program in order to ascertain sustainable effects.

## A Third Glance: Emerging Parallels to Prevention of Cardiovascular and Infectious Diseases

The disappointment at prevention in the past and the cautious curiosity about many new trends in current prevention contrast with their prospects in the future. There are many reasons for optimism. The first is a simple backlog effect: given that psychiatry – including prevention and other domains of public mental health – distinctly lags behind most domains of somatic medicine, it follows that much remains to be developed and to be done. The burning question, however, is when the next transition in psychiatry will start.

More and more signs are emerging that the next move is drawing closer. Among these signs are the increasingly recognized comorbidities and other parallels with somatic diseases, the next component of the four-field health scheme (see Figure 1). The manifold associations between psychiatric and allergic diseases are perhaps the most striking links (23, 24), while the links to CVDs are certainly the best-known ones (25).

Apart from comorbidity measures, common risk factors such as obesity and poor diet, smoking, and physical inactivity also strengthen the link between CVD and common mental disorders (CMD) such as depression [for an overview see Ref. (26)]. The most probable common denominator is systemic inflammation and immune dysregulation (27). Jacka and Berk have emphasized the idea that most of the prevention done within the context of CVD encompasses in principle also common in mental disorders such as depression. Thus, prevention related to life-style factors emerges as a new pathway, not only in CMD but also in schizophrenia (28) and, possibly, other mental disorders as well. For example, the intake of omega-3 fatty acids is a current subject of trials (29). A particularly interesting side-effect is that life-style factors open the gate to universal prevention.

Another challenging pathway for universal prevention emerges from the associations between psychiatric and autoimmune diseases (30–32). There is even growing evidence that some psychiatric diseases – for example, some subtypes of OCD and ADHD – are autoimmune diseases in the narrow sense (33).

Autoimmunity is typically related to an infectious disease etiology. As a matter of coincidence, it is foreseeable from upcoming research that infectious diseases are important risk factors in mental disorders (34, 35). Thus, many risk factors and markers gradually emerge in a different light: the social class gradient, familial aggregation, sex-ratios, etc. Equally, many opportunities for prevention are appearing on the horizon which are similar to those in somatic medicine – and no less promising: hygiene, vaccines, appropriate treatment of noxious microbes, etc.

## A Fourth Glance: Prevention in Psychiatry Will Work Better than Prevention in Somatic Medicine

While prevention of psychiatric diseases will presumably grow closer to prevention in somatic medicine, it will nonetheless retain genuine advantages. First, given that psychiatric diseases typically emerge from two or even more “hits” (36), there must also be several opportunities for prevention to intervene and modify the risk of definitively developing a psychiatric disease or more serious symptoms.

Second, the etiopathogenesis of psychiatric diseases obviously involves age stages of enhanced vulnerability. It is deducible from the age at onset parameters that the “multiple hits” occur not only in different combinations but also during different age stages. Better knowledge of biologically and psycho-socially vulnerable age stages and the risk mechanisms involved will help to focus and optimize preventive efforts.

Third, the notion that the autoimmunity link applies for some subtypes of ADHD or OCD directs attention to the subtyping of existing psychiatric diagnoses. It is foreseeable that progress in research and advances in subtyping will lead not only to thicker diagnostic manuals but also to more specific information about specific disease characteristics, brain networks involved, risk factors, etiopathogenic concepts, vulnerable age stages. Subtyping will finally lead to prevention strategies that may be better tailored to specific diseases and risk groups.

There are even more promising prospects. A particular characteristic of psychiatric diseases is that they are biologically and psycho-socially driven. Complementarily, many therapies are most promising if they combine drugs, behavioral modification, and psychotherapy. Strikingly, all these avenues of action are also open for prevention, and will finally include the fourth and last component of the four-field scheme, positive mental health (see Figure 1). Hopefully, unambiguous concepts (37) and instruments (38, 39) with respect to positive mental health will be developed in the near future, capable to fulfill the precondition of being more than a reciprocal proxy of mental illness. However, this challenge is incomparably more complicated than in somatic health.

In sum, the next transition in psychiatry has probably already started. Psychiatry can be expected to change considerably in the next 10–15 years, and prevention will benefit disproportionately from its progress. It is even imaginable that prevention in psychiatry will have more tools and will work better than prevention in somatic medicine.

## Conflict of Interest Statement

The author declares that the research was conducted in the absence of any commercial or financial relationships that could be construed as a potential conflict of interest.
